# Redundancy-Based Validity Screening of Strain-Gauge Records from In-Service Hydraulic Turbine Servomotors

**DOI:** 10.3390/s26216712

**Published:** 2026-10-23

**Authors:** Hong Hua, Xiaobing Liu, Wanquan Deng

**Affiliations:** Key Laboratory of Fluid and Power Machinery, Ministry of Education, Xihua University, Chengdu 610039, China

**Keywords:** strain gauge, sensor validation, analytical redundancy, guide-vane servomotor, hysteresis, condition monitoring

## Abstract

**Highlights:**

- A redundancy-based validity screen refused none of 15 reference strain-gauge configurations but rejected one unsound servomotor record.
- Channel configuration at one actuator shifted the cross-actuator variation in the dissipation index from 6.6% to 20.8%.
- Field hysteresis and dissipation data should be reported only after every strain-gauge record has been screened for validity.
- A fourth gauge forming a second opposed pair lets bending be distinguished from gauge faults at a rod section.

**Abstract:**

Strain gauges on the push–pull rod are a practical means of measuring the load on in-service hydraulic actuators, but the channels degrade silently, and a degraded channel still yields a credible-looking load characteristic. A redundancy-based validity screen for such records is presented and applied to every record from four guide-vane servomotors of Francis units under wind–photovoltaic–hydro complementary dispatch. Four elementary screens are combined: channel coherence with an applicability floor, configuration sensitivity of the interval loading gradient against a tolerance band built from fifteen reference channel configurations, step magnitude, and abscissa integrity. The screen refuses none of the reference configurations, in sample or under leave-one-out. At a three-channel actuator it localises a disagreement to one gauge; at a two-channel actuator it can only detect one, and it refuses a record whose loading gradient, −310 kN per unit relative opening, lies 365 kN below the reference range. For records that pass, loop energy is expressed through a stroke-free dissipation index and a growth rate. The channel configuration carried forward at a single actuator moves the cross-actuator coefficient of variation in the index between 6.6% and 20.8%, whereas the grouping by growth rate is preserved for every configuration that excludes the isolated gauge.

## 1. Introduction

The large-scale integration of wind and photovoltaic generation has changed what a hydropower unit is asked to do. In complementary portfolios the hydro plant absorbs the residual variability of the other resources, and coordinated dispatch studies treat it as the fast balancing resource [1,2]. The mechanical cost of that role is governed by the number of regulating movements rather than by the energy delivered [3,4], and regulation quality and machine loading must be assessed jointly under wide-range operation [5]. Within the unit, a large share of this burden is carried by the governing system, whose duty is dominated by small, frequent corrections and whose failure modes under high-cycle regulation service have been catalogued for small-to-medium units [6,7]. Condition monitoring of hydro and pump-turbine units has nonetheless been directed mainly at the rotating assembly and the water passage rather than at the regulating actuator [8,9].

The guide-vane servomotor is where the governor command becomes a mechanical displacement. Strain gauges bonded to its push–pull rod can be installed on an operating machine without modifying the hydraulic circuit, and they have been used, together with finite-element analysis, to predict the fatigue life of the rod and its connections [10]. The same measurement, recorded against the governor feedback signal, yields a hysteresis loop in the load–opening plane. The loop is familiar from the control literature, where it appears as a nonlinearity to be compensated in speed-governor and position-servo design [11,12,13], and its constitutive description is well developed through static and dynamic friction models and hysteresis operators [14,15,16,17,18,19]. The area it encloses is work dissipated in the load path, which makes it a candidate indicator of the mechanical cost of regulation service.

Using field strain records for this purpose raises a measurement problem before it raises a mechanical one. Gauges bonded to a rod that reverses continuously in an oil-wetted environment degrade in service, and a degraded channel does not fail conspicuously: it continues to return dimensionally plausible values, which enter the loop construction indistinguishably from mechanical information. Sensor validation based on analytical redundancy addresses exactly this situation [20,21,22]: data anomaly detection has become a standard stage of structural health monitoring pipelines [23], fault-tolerant control schemes reconstruct faulty sensor signals in electro-hydraulic servo systems [24], and redundancy-based fault detection has recently been demonstrated for axial deformation sensors on hydraulic turbine head-cover bolts [25]. These methods, however, rely on continuous time series from which residual statistics can be estimated. Field campaigns on operating units frequently archive only reduced quasi-static load–opening points, a few per branch, which do not support such statistics. Uncertainty evaluation does not close the gap either: the GUM framework and its Monte Carlo supplement quantify the dispersion of a measurement that was actually made [26,27,28], but they cannot reveal that a channel has stopped reporting the intended quantity, which is the task of sensor fault diagnosis [29].

Two further features of the field situation shape the problem. First, the physical stroke corresponding to the governor feedback signal is generally not calibrated, so any loop energy expressed in joules rests on an unmeasured quantity. Second, three gauges at one cross-section of a rod subject to eccentric loading provide less redundancy than their number suggests, because a general bending field introduces two unknowns in addition to the axial strain. What such a set can and cannot decide must therefore be stated rather than assumed.

This paper develops and exercises a validity screen for reduced strain-channel records from in-service hydraulic servomotors, using four guide-vane servomotors of Francis units under complementary dispatch. The contributions are threefold. (i) A screen of four elementary checks, covering both axes of the loop, is defined and applied symmetrically to every record, including those believed to be sound, so that its specificity on reference data is measured rather than asserted. (ii) The limits of the screen are made explicit: three channels permit a disagreement to be localised whereas two permit it only to be detected, and at a 0°/90°/180° gauge layout a localised disagreement cannot be allocated between a gauge fault and a bending field without further information. (iii) For records that pass, a stroke-free dissipation index and a dissipation growth rate are defined, and the sensitivity of cross-actuator conclusions to the channel configuration carried forward is quantified. The strain records were acquired in the field campaign described in [10], where the record of S1 was used to predict the fatigue life of the rod and its connections; the data reused from that work and the elements that are new here are stated explicitly in Section 2.1. Section 2 describes the units, the measurement chain and the methods; Section 3 reports the results; Section 4 discusses their interpretation and limitations; and Section 5 concludes. The symbols are listed in the Nomenclature at the end of the paper.

## 2. Materials and Methods

### 2.1. Units and Data Provenance

Four Francis units at small-to-medium run-of-river stations in western Sichuan, China, were instrumented. All four are dispatched within a regional wind–photovoltaic–hydro complementary portfolio under automatic generation control, with the governor in opening-feedback mode, so the guide-vane actuator receives small corrective commands through most of the operating day. The actuators are identified by the codes S1–S4, and station names and locations are not given. Table 1 summarises the units; S1 is the unit whose servomotor was characterised for fatigue in [10], where its further design parameters are reported. Rated heads range from 91 to 132 m and rated capacities from 12.0 to 20.6 MW, and the instrumented rods range from 70 to 102 mm in diameter, a factor of 2.1 in cross-sectional area. The rod diameters are consistent with the archived records: at every recorded point the reported load equals the mean of the reported channel stresses multiplied by the nominal cross-section to within 35 N, the resolution implied by the two-decimal rounding of the archived stresses.

From [10], only the description and characterisation of the measurement chain, the design parameters of S1 and the load–opening record of S1 are reused; there, that record served for fatigue-life prediction and a full-traverse hysteresis characterisation, without examination of individual channels. The records of S2–S4 are reported here for the first time, and the validity screen, the interval and stroke-free descriptors, their uncertainty evaluation and the resulting findings are new to this paper. Fault histories compiled for these stations [6] are used only as background when the screening verdicts are discussed; they are historical records and are not contemporaneous with the tests.

### 2.2. Measurement Path and What the Rod Load Represents

**Figure 1:**
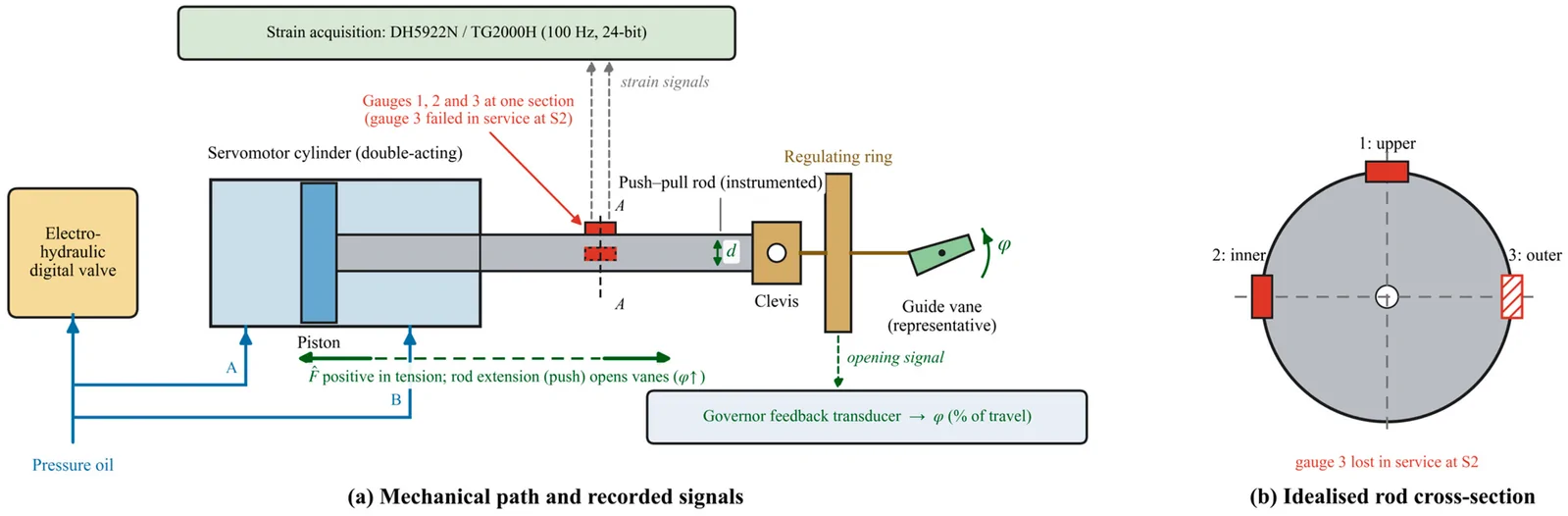
Instrumented guide-vane servomotor. (**a**) Mechanical path and recorded signals; the gauged section A–A lies on the rod between the cylinder head and the clevis. (**b**) Idealised rod cross-section at A–A showing gauge positions 1 (upper), 2 (inner, towards the main shaft) and 3 (outer); gauge 3, lost in service at S2, is hatched.

Figure 1a shows the path from governor command to guide vanes. Pressure oil is admitted to a double-acting cylinder through an electro-hydraulic digital valve; the piston drives a push–pull rod connected through a clevis to the regulating ring, whose rotation turns all guide vanes in unison. At S1 the regulating ring is driven by two servomotors [10], and where a ring is driven by more than one servomotor the instrumented rod carries its own share of the actuating force; since every descriptor below is evaluated per actuator and compared either with the same actuator or through dimensionless or configuration-based quantities, the analysis does not depend on that share. The sense of the linkage is fixed by the measurements. At every actuator the rod load on the opening traverse exceeds that on the closing traverse at the same opening over the comparison interval, and at the three reference actuators the rod is in tension at every recorded point of the opening traverse, so the rod retracts and pulls the ring in the opening direction. The rod load, positive in tension, is denoted $\hat{F}$, the circumflex marking a nominal load, i.e., one computed from an assumed elastic modulus and a nominal cross-section and not calibrated in situ.

The position of the gauged section determines what the measured loop contains. Section A–A lies outside the cylinder, between the rod gland and the clevis. In the quasi-static regime of the tests, in which the rod moves at a mean velocity of the order of 1 mm/s, the axial force at this section equals the force transmitted to the regulating ring, that is, the hydraulic torque on the guide vanes reflected through the linkage, together with the friction in the regulating-ring guides and wear pads, the guide-vane stem bushings, the link pins and any overload-protection coupling in the linkage. Friction in the piston and rod seals acts between the cylinder and the piston–rod assembly on the cylinder side of the section and does not pass through it. The hysteresis loop in $\hat{F}$ therefore characterises the driven mechanism and the vane hydraulic loading, not the seals of the servomotor itself; a measurement of the total actuator friction would require the chamber pressures in addition.

The abscissa is the guide-vane opening φ reported by the governor feedback transducer, in per cent of actuator travel. It is the signal against which the machine is actually controlled, but it is not a calibrated displacement: the rod travel corresponding to one per cent of φ was not measured. Every result below is therefore expressed in φ or in a form invariant to the missing stroke calibration. The feedback transducer is itself an in-service instrument and is therefore screened together with the strain channels (Screen D below) rather than assumed sound.

Three uniaxial strain gauges were bonded at section A–A, oriented along the rod axis, at the circumferential positions shown in Figure 1b: gauge 1 upper, gauge 2 inner and gauge 3 outer, that is, at approximately 90°, 180° and 0° about the rod axis. At S2, gauge 3 failed in service before the recorded manoeuvres, leaving the pair {1, 2}; S1, S3 and S4 retained all three channels.

### 2.3. Acquisition and Test Protocol

Strain signals from all available channels, measured with BX120-3AA foil gauges (120 Ω, gauge factor 2.08 ± 1%, 3 mm grid) [10], were acquired synchronously with a DH5922N dynamic signal acquisition system with TG2000H strain conditioning at 100 Hz and 24-bit resolution, and the governor feedback signal was recorded on the same time base. All four units were in scheduled service and connected to the grid at the time of test, and the opening was changed through the load set-point, which is consistent with the opening-dependent hydraulic loading evident in the mid-line of the load–opening loops. Grid-connected load-change tests were carried out on the same units, raising the load in four steps and lowering it in four steps; at S1, for example, the active power was raised from 1.3 to 17.9 MW and returned to 1.2 MW. The characterisation of the same strain chain in [10] established a zero drift within ±2 µε after 24 h of stabilisation and a temperature coefficient within ±0.5 µε/°C, which for E = 206 GPa corresponds to about ±0.4 MPa and ±0.1 MPa/°C. Bridge settings were fixed for each actuator’s campaign, and no channel was rebalanced between the opening and closing manoeuvres, since a rebalance between branches would introduce a step into the loop that could not afterwards be distinguished from a mechanical one. No quantity used for screening or for dissipation reporting depends on the state in which the bridges were balanced, because a constant bridge offset cancels from every branch difference and every gradient, and absolute load levels are accordingly not used for inference.

At each unit the actuator was taken through one complete opening manoeuvre followed by one complete closing manoeuvre, over as much of the travel as plant operating constraints permitted. The records were reduced to load–opening points at successive openings between 2% and 10% of travel apart, giving 99 points in total across the eight branches. The reduced strain archive does not retain a continuous time series. Mean traverse rates were therefore estimated by dividing each traversed opening range by the summed step durations of the corresponding direction of the load-change tests, which assumes that the recorded range was traversed during those tests: 0.25%/s on opening and 0.20%/s on closing at S1, 0.21%/s and 0.20%/s at S3, and 0.46%/s and 0.58%/s at S2; no load-change timing is available for S4. These rates are indicative and are used only to compare actuators. For strokes plausible for machines of this class, these rates correspond to a mean rod velocity of the order of 1 mm/s, at which inertial and oil-column dynamic contributions to the rod load are negligible. Friction in lubricated contacts can nevertheless vary appreciably with velocity at such speeds [30,31,32], so all separations reported here are total separations at the stated traverse rate. No manoeuvre was repeated, so a direct repeatability estimate is not available; the potential impact of this on the reported values is bounded in Section 2.9.

### 2.4. Nominal Rod Load, Bending and the Redundancy Assumption

Each retained channel *i* returns an archived rod stress from the measured axial surface strain,

*σ_i_* = *Eε_i_*, *i* ∈ *C* (1)

where *E* is the elastic modulus of the rod material and *C* is the set of channels used: *C* = {1, 2, 3} at S1, S3 and S4 as acquired, and *C* = {1, 2} at S2. The nominal axial stress is the mean over *C* and the nominal rod load follows from the cross-section *A* = *πd*^2^/4,

(2) $$\bar{\sigma }=\frac{1}{\left|C\right|}\sum_{i\in C}\sigma_{i},\;\hat{F}=A\bar{\sigma }$$

With the gauges on the rod surface at circumferential positions *θ* = 0° (gauge 3), 90° (gauge 1) and 180° (gauge 2), a rod of diameter *d* carrying axial strain *ε*_0_ and a small bending field with curvature components *κ_x_* and *κ_y_* has, to first order, the surface strain

(3) $$\varepsilon (\theta )=\varepsilon_{0}+\frac{d}{2}\left(\kappa_{y}\cos\theta +\kappa_{x}\sin\theta \right)$$

The diametrically opposed pair {2, 3} therefore returns *ε*_0_ exactly; the three-channel mean returns *ε*_0_ + *κ_x_d*/6; and the orthogonal pairs {1, 2} and {1, 3} return *ε*_0_ + (*κ_x_* − *κ_y_*)*d*/4 and *ε*_0_ + (*κ_x_* + *κ_y_*)*d*/4, retaining both curvature components at half weight. The exact cancellation holds only for exact gauge placement, and only the nominal positions were recorded.

Equation (3) also bounds what the gauge set can decide. Three gauges and three unknowns (*ε*_0_, *κ_x_*, *κ_y_*) form an exactly determined system, so any disagreement between channels can in principle be absorbed by a bending field, and there is no redundancy for fault detection without an additional assumption. The assumption adopted here is that the bending field arises from an approximately constant eccentricity of the load path, so that the channel stresses co-vary with the axial load. The validity screens defined below test consistency with this assumption. A refusal therefore means that the record is inconsistent with a single axial load accompanied by a load-proportional bending field; it may arise from a channel fault or from a bending field that varies independently of the axial load, and the two are distinguished only where further evidence is available.

The modulus *E* is assumed rather than measured, *A* is a nominal area, and no in situ calibration of the assembled rod was possible. The relation between the reported and the true rod load *F* is written

(4) $$\hat{F}=\mathrm{cF}+b+\eta$$

with *c* a multiplicative scale error, *b* an offset fixed by the state in which the bridges were balanced, and *η* a zero-mean random component. Because the bridges were balanced once per actuator, *b* is constant within a record and cancels identically from every descriptor built from branch differences or gradients with respect to opening. It does not cancel from absolute load levels, including the level of the midline *R* defined below and the opening at which it crosses zero; those quantities are interpretable only if the bridges were balanced with the rod unloaded. The scale error *c* multiplies every load-dimensioned descriptor and cancels from dimensionless ones.

### 2.5. Branch Construction and Hysteresis Descriptors

Each record yields an opening branch ${\hat{F}}_{o}(\varphi )$ and a closing branch ${\hat{F}}_{c}(\varphi )$. Because *φ* is recorded rather than commanded, the two branches are linearly interpolated onto a common uniform grid with a step of 0.01% over the comparison interval; linear interpolation is preferred because a smoothing spline would attenuate the abrupt local features that the screen is designed to detect. The branch separation and the midline are

(5) $$2f(\varphi )={\hat{F}}_{o}(\varphi )-{\hat{F}}_{c}(\varphi ),\;R(\varphi )=\frac{1}{2}\left[{\hat{F}}_{o}(\varphi )+{\hat{F}}_{c}(\varphi )\right]$$

The separation is the additional load required to hold a given opening when approached from below rather than from above, with *f* the half-width of the loop in the Coulomb-type notation [14]. The mid-line plays the role of the backbone curve in hysteretic modelling [18,33]: it approximates the load that would remain if the direction-dependent resistance were removed, under the approximation that this resistance is of equal magnitude in the two directions. Its interval least-squares gradient with respect to *y* is denoted *R*′.

Three descriptors are evaluated on the comparison interval [*φ*_1_, *φ*_2_] of width Δ*φ*. The signed normalised separation expresses the mean separation as a fraction of the load excursion of the loop,

(6) $$H_{D}=\frac{\left\langle 2f(\varphi )\right\rangle }{\max\left({\hat{F}}_{o},{\hat{F}}_{c}\right)-\min\left({\hat{F}}_{o},{\hat{F}}_{c}\right)}$$

where ⟨·⟩ denotes the interval mean and the extrema are taken over both branches pooled. *H*_D_ is dimensionless and independent of *b* and *c*. A positive value indicates an opening branch lying above the closing branch on average, as expected of a dissipative loop; a negative value indicates branch inversion, which is treated as a diagnostic flag rather than as a machine property.

So that all gradients carry SI-consistent units, they are taken with respect to the relative opening *y* = *φ*/100, which is dimensionless and runs from 0 to 1 over the full travel; the comparison interval [*φ*_1_, *φ*_2_] then corresponds to [*y*_1_, *y*_2_] of width Δ*y* = Δ*φ*/100. The interval loading gradient is the least-squares gradient of the opening branch with respect to *y*,

(7) $$K_{D}\equiv K_{o}=\frac{d{\hat{F}}_{o}}{dy},\;y=\frac{\varphi }{100}\in \left[y_{1},y_{2}\right]$$

*K*_D_, in kN, is the change in rod load that a full-travel traverse would produce at the interval gradient (a value of 100 kN corresponds to 1 kN per percentage point of opening); it describes the direction in which the load path is loaded as the vanes open. Over the part-load band, the rod load of these units is expected to rise with opening, but the hydraulic torque characteristic of a guide-vane cascade need not be monotonic, so a negative *K*_D_ is not in itself physically excluded. *K*_D_ is therefore used in the validity screen (Screen B) as a screening quantity by comparison with a reference population, not as a test of physical possibility.

The branch crossing count *N*_x_ is the number of grid openings within the interval at which the sign of 2*f*(*φ*) changes. For a conventionally traversed loop *N*_x_ = 0, and a non-zero value indicates inversion or a local feature large enough to reverse the separation.

### 2.6. Stroke-Free Dissipation Indices

The work dissipated in one traverse of the loop over rod displacement *x* is *W* = ∮*F* d*x*. The displacement was not recorded, but it is related to the relative opening by the unknown full stroke *L* through *x* = *yL*, so that

(8) $$W=L\oint \hat{F}\;dy=LA_{L},\;A_{L}=\int_{y_{1}}^{y_{2}}2f\;dy$$

where *A*_L_, the interval loop area in the plane of load and relative opening, is obtained by trapezoidal integration of the interpolated separation. The stroke enters the energy as a single multiplicative constant and can therefore be factored out rather than assumed. The stroke-free dissipation index

(9) $$w_{L}=A_{L}=\frac{W}{L}$$

is the energy dissipated per metre of full actuator stroke, in kJ/m (numerically equal to *A*_L_ in kN), and becomes an absolute energy, *W* = *w*_L_*L*, whenever a stroke is supplied. Two restrictions apply. The index is an interval area for a traverse of the whole comparison interval, not a full-cycle area, and it does not describe the minor loops traced by small regulating corrections within the interval. It also inherits the scale error *c* in full: the constant offset *b* cancels from the branch separation, whereas *c* multiplies it, so a relative error in *c* appears as the same relative error in *w*_L_ (a chain gain 2% too high returns a value of *w*_L_ 2% too high). Values of *w*_L_ at different actuators can therefore be compared only to within the combined scale uncertainty of their two chains (Section 2.9).

**Figure 2:**
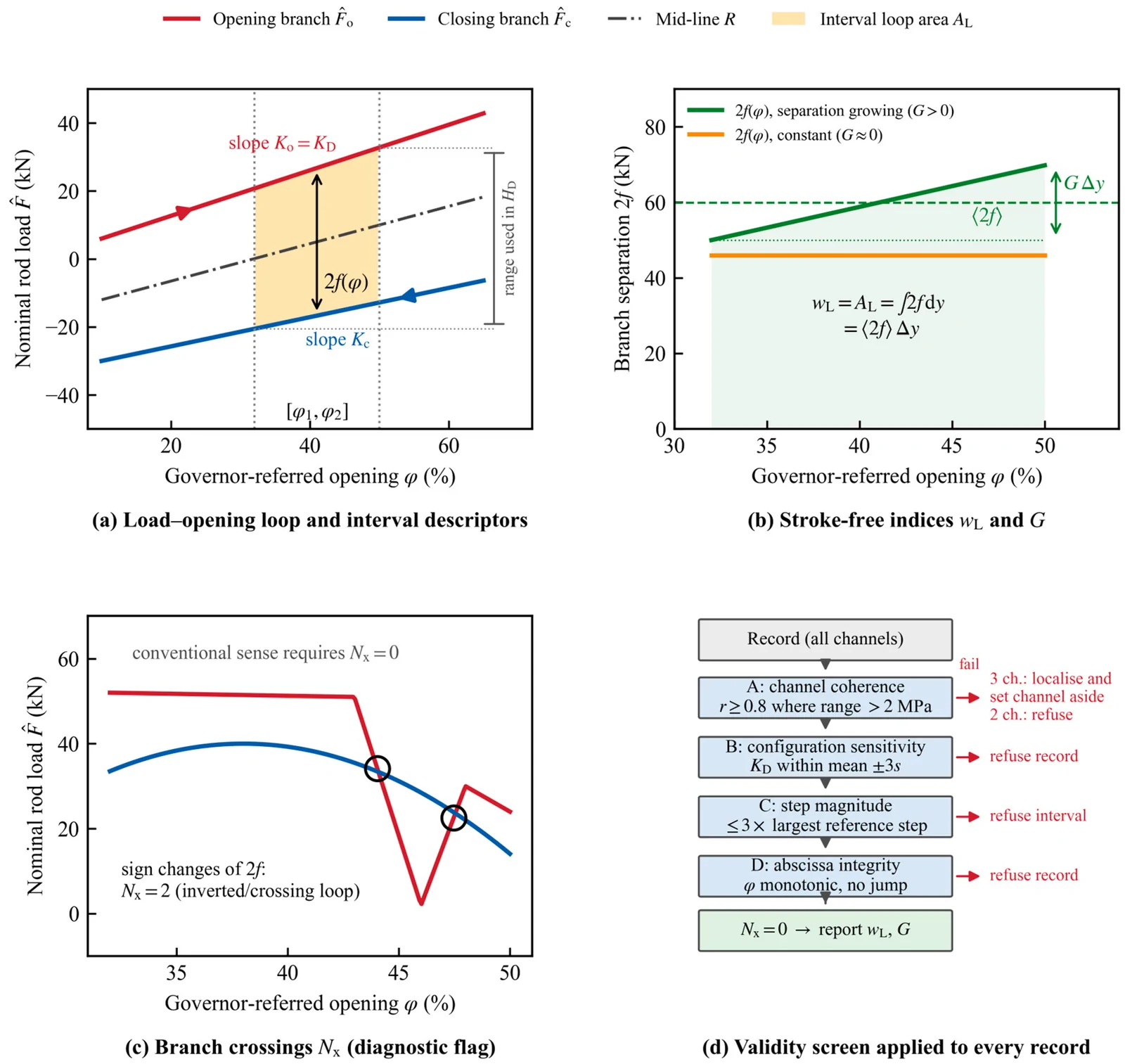
Schematic of the descriptors, indices and validity screen (illustrative values only). (**a**) Idealised load–opening loop traversed in the conventional, clockwise sense (opening branch above, traversed towards larger opening; closing branch below, traversed back), with mid-line *R*, separation 2*f*(*φ*), branch gradients *K*_o_ = *K*_D_ and *K*_c_ on the comparison interval [*φ*_1_, *φ*_2_], interval loop area *A*_L_ (shaded) and the pooled load range that normalises *H*_D_. (**b**) Branch separation across the interval: *w*_L_ is the area under 2*f* in relative opening *y* and *G* is its slope, so a separation that grows across the interval and one that is constant are distinguished by *G*. (**c**) A record whose branches cross (*N*_x_ = 2), for which the loop area is a net signed quantity and the indices are not defined. (**d**) Sequence of Screens A–D applied to every record before the indices are reported.

Loop area does not distinguish an actuator whose separation is constant across the interval from one whose separation grows across it. Since ${\hat{F}}_{o}$ = *R* + *f* and ${\hat{F}}_{c}$ = *R* − *f*, a second index follows from the branch gradients,

(10) $$G\equiv \frac{d\left(2f\right)}{dy}=2\left(K_{o}-R\prime \right)=K_{o}-K_{c}$$

where *K*_c_ is the closing-branch gradient on the same interval and *G*, like *K*_o_ and *K*_c_, is expressed in kN; it is the growth of the separation that a full-travel traverse would produce at the interval gradient. The right-hand form is used throughout, so both gradients are always evaluated on the same window. *G* ≈ 0 indicates a position-independent resistance, and a large positive *G* a resistance that rises with opening. Both indices presuppose *N*_x_ = 0: where the separation changes sign, the integral in Equation (8) is a net signed area in which positive and negative contributions cancel, and the midline no longer separates two non-intersecting branches. The descriptors and indices defined in Section 2.5 and Section 2.6, and the screen that precedes their use, are illustrated schematically in Figure 2.

### 2.7. Common Comparison Interval

A mean separation, a least-squares gradient and a loop area are all interval quantities, so values computed over different intervals are different quantities. The comparison interval is fixed as the intersection of the opening ranges actually traversed (Table 1): approximately 13–63% at S1 and S3, 32–83% at S2 and 14–50% at S4, so that

[*φ*_1_,*φ*_2_] = [32%, 50%], Δ*φ* = 18%, Δ*y* = 0.18 (11)

The interval is determined by plant operating constraints on the days of test and not by the descriptor values, and it lies within the part-load band in which regulating traffic concentrates. Its narrowness makes interval gradients sensitive to the scatter of the four to six points available per branch; this is addressed through the Type A standard uncertainties of the fits and through a window-sensitivity analysis reported with the results. At S4 the closing branch ends at 49% and is held at its last recorded value over the last 1% of the interval.

### 2.8. Redundancy-Based Validity Screen

The screen decides, before any descriptor is interpreted, whether a record is fit to be interpreted. Four screens, A to D, are applied in sequence, and a record must pass all four before dissipation indices are reported for it. The screens are deliberately elementary. Principal-component, parity-space and observer-based residual tests [20,21,22,24] would be preferable where continuous time series are available, but the archived records consist of a few reduced points per branch, which is too few to estimate the residual statistics those methods require. Two properties of the screen should be stated at the outset. It establishes whether a record is consistent with a reference population, not whether a machine is healthy, and a degradation shared by all reference actuators would pass undetected. And it only removes data: no imputation or reconstruction of a refused channel is attempted, since any such repair would import the assumption that the screen exists to test. The screens and their criteria are summarised in Table 2.

For Screen A, *r* is computed on six to thirteen points per segment and is used as a descriptive measure of common trend, not as a significance test. The threshold of 0.8 rejects gross loss of common behaviour rather than enforcing agreement; its basis is given below. The applicability floor of 2 MPa, about 10 µε and five times the zero-drift bound of the chain, ensures that the common signal dominates resolution and drift; segments below it are not judged. With three channels a single discordant channel appears in the two pairs that contain it while the third pair remains coherent, so the disagreement is localised; with two channels it can only be detected. Configurations containing a localised channel are excluded from the reference population of Screen B. The sensitivity of the outcome to the threshold and the floor is reported with the results.

The threshold is set from the coherence expected of two intact channels at the applicability floor, not tuned to the data. Under load-proportional bending, two intact channels are linear functions of the same axial load and would return *r* = 1 in the absence of noise, whatever their relative gain. The dominant disturbance that the reduced archive cannot remove is a slow drift bounded by the zero-drift specification of the chain, ±0.4 MPa. Treating this bound as a rectangular distribution (standard deviation 0.23 MPa) and the common signal as a linear trend spanning the 2 MPa floor (standard deviation 2/√12 ≈ 0.58 MPa), the expected correlation of two intact channels is 0.58^2^/(0.58^2^ + 0.23^2^) ≈ 0.86. A threshold of 0.8 therefore lies just below the lowest coherence expected of intact channels at the floor, and its stringency increases as the stress range grows. Because *r* is computed on only six to thirteen points, a single observation can move it appreciably: the 95% confidence interval obtained from Fisher’s *z*-transformation is [0.43, 0.99] for *r* = 0.92 on six points and [0.45, 0.94] for *r* = 0.80 on thirteen points. This is why *r* serves only as a descriptive measure and why the verdicts are checked against a sweep of the threshold and the floor and against a leave-one-point-out recomputation. Pearson’s *r* is moreover insensitive by construction to a gain error between channels. Screen A is therefore aimed at loss of common behaviour, such as debonding, drift, sign reversal or intermittent contact, whereas gain errors are left to Screen B, in which a gain error in one channel shifts *K*_D_ of every configuration that contains that channel. A gain error small enough to pass both screens changes the reported descriptors only in proportion to its size: a 10% gain error in one of three channels moves the three-channel mean by about 3%, which is comparable with the Type B scale uncertainty of the chain.

For Screen B, the mechanics at each reference actuator is fixed, so recomputing *K*_D_ from subsets of its channels isolates the effect of channel configuration; after the exclusion above, fifteen configurations remain, six at S1, three at S3 and six at S4. A minimum-to-maximum envelope is not used, because its extremes are defined by two of its own members and would always be rejected under leave-one-out. The configurations at one actuator share its mechanics and most of its measurement chain, so the band is a heuristic envelope rather than a calibrated confidence region, and distances from it are reported in kN rather than in standard deviations. For Screen C, two populations are formed from the three channel pairs at the reference actuators and kept apart: the change that a pair configuration induces in a recorded step, which bounds what channel configuration alone can do, and the magnitude of the steps themselves, which bounds what intact operation produced and defines the criterion. For Screen D, a slipped linkage or a discontinuous position signal would place correct loads at incorrect openings and mimic a strain-side fault; cross-checks against the commanded opening and the unit power would strengthen this screen but require continuous time series.

### 2.9. Uncertainty Evaluation

Uncertainty evaluation and validity screening answer different questions: an uncertainty statement quantifies the dispersion of values attributable to a measurement that was actually made [26], and a coverage interval computed from a degraded channel correctly describes the dispersion of an incorrect quantity. The Type B components of the strain-to-load conversion are those established for strain-gauge chains [27,28], dominated by the tolerances on the gauge factor, *E* and *A*, which together constitute the scale error *c*. The calibration records required for an actuator-specific evaluation of *u*(*c*) were not retained, and *u*(*c*) is therefore estimated from nominal specification limits treated as rectangular distributions: ±1% on the gauge factor, as specified for the BX120-3AA gauges [10]; ±3% on *E* about the value of 206 GPa specified for structural steel in GB 50017-2017 [34], a bound that covers the range of about 200–212 GPa of carbon and low-alloy rod steels; ±0.2% on *A*, corresponding to an f8 fit tolerance on rod diameters of 70–102 mm; and a conservative ±0.5% on the gain of the conditioning and acquisition chain, which exceeds the ±0.1% stated for the TG2000H conditioning [10]. The modulus dominates the budget. These give *u*(*c*) ≈ 1.9% (*k* = 1), or an expanded uncertainty of about 3.7% (*k* = 2), for every load-dimensioned quantity of one actuator, and about 2.6% for the ratio of such quantities at two actuators with independent chains. The conclusions of this paper are accordingly drawn from quantities in which *c* cancels: *H*_D_, *N*_x_ and the ratio *G*Δ*y*/⟨2*f*⟩ are independent of *c*, and each comparison between channel configurations at one actuator shares that actuator’s value of *c*. Absolute values of *K*_D_, *K*_c_, *G* and *w*_L_ scale with *c*, and differences between actuators smaller than the combined scale uncertainty of their two chains cannot be resolved. The Type A standard error of each interval gradient is obtained from the residuals of its least-squares fit; these values are lower bounds on the combined uncertainty and are labelled as such.

Two further components are evaluated. The zero drift of the chain, bounded by ±0.4 MPa over 24 h, cancels from every gradient because it is common to all points of a branch, but a drift occurring between the opening and closing manoeuvres would offset one branch against the other and enter the separation directly. Multiplied by the rod cross-sections, the bound corresponds to ±1.5 kN at S3, ±2.0 kN at S1, ±2.5 kN at S2 and ±3.3 kN at S4, that is, to at most 2–6% of the interval mean separations; as a 24 h bound it is conservative for manoeuvres lasting minutes, and it is included as a rectangular component in the uncertainty of *w*_L_. The Type A standard uncertainty of each branch gradient, *K*_o_ or *K*_c_, is obtained from the residuals of its least-squares fit to the recorded points on the interval, and that of the interval mean of each branch from the same fit; the uncertainties of *G* and ⟨2*f*⟩ follow by combining the two branches in quadrature, and that of *w*_L_ from ⟨2*f*⟩ and Δ*y*. The sensitivity of every descriptor to individual observations is examined separately by a leave-one-point-out (jackknife) recomputation, in which each recorded point inside the interval, or adjacent to it and used in the interpolation, is removed in turn. The combined standard uncertainties (*k* = 1) are reported with the values in Table 3 and Table 4; they do not include the repeatability of the manoeuvre.

The potential impact of the missing repeatability estimate can nevertheless be bounded by distinguishing two kinds of results. Comparisons between channel configurations of one record, which include every screening verdict and the configuration dependence of *w*_L_, are unaffected, because all configurations share the same traverse and therefore the same run-to-run deviation. Comparisons between actuators are affected, for instance through the velocity dependence of friction in lubricated contacts [30,31,32]. As an illustration, an independent run-to-run variation of ±5% in *w*_L_ at each actuator would by itself move the cross-actuator coefficient of variation in the three-channel values (6.6%) anywhere between 1.9% and 12.0%, and a variation of ±10% would move it between 1.8% and 17.5%. For the growth rate, a repeat would have to change the growth of the separation across the interval by about 18 kN, roughly one third of the mean separation, to remove the distinction between S1 and the other two actuators. Cross-actuator differences are therefore interpreted only where they exceed these bounds, and the coefficients of variation reported below are single-traverse values.

## 3. Results

### 3.1. Loop Morphology

**Figure 3:**
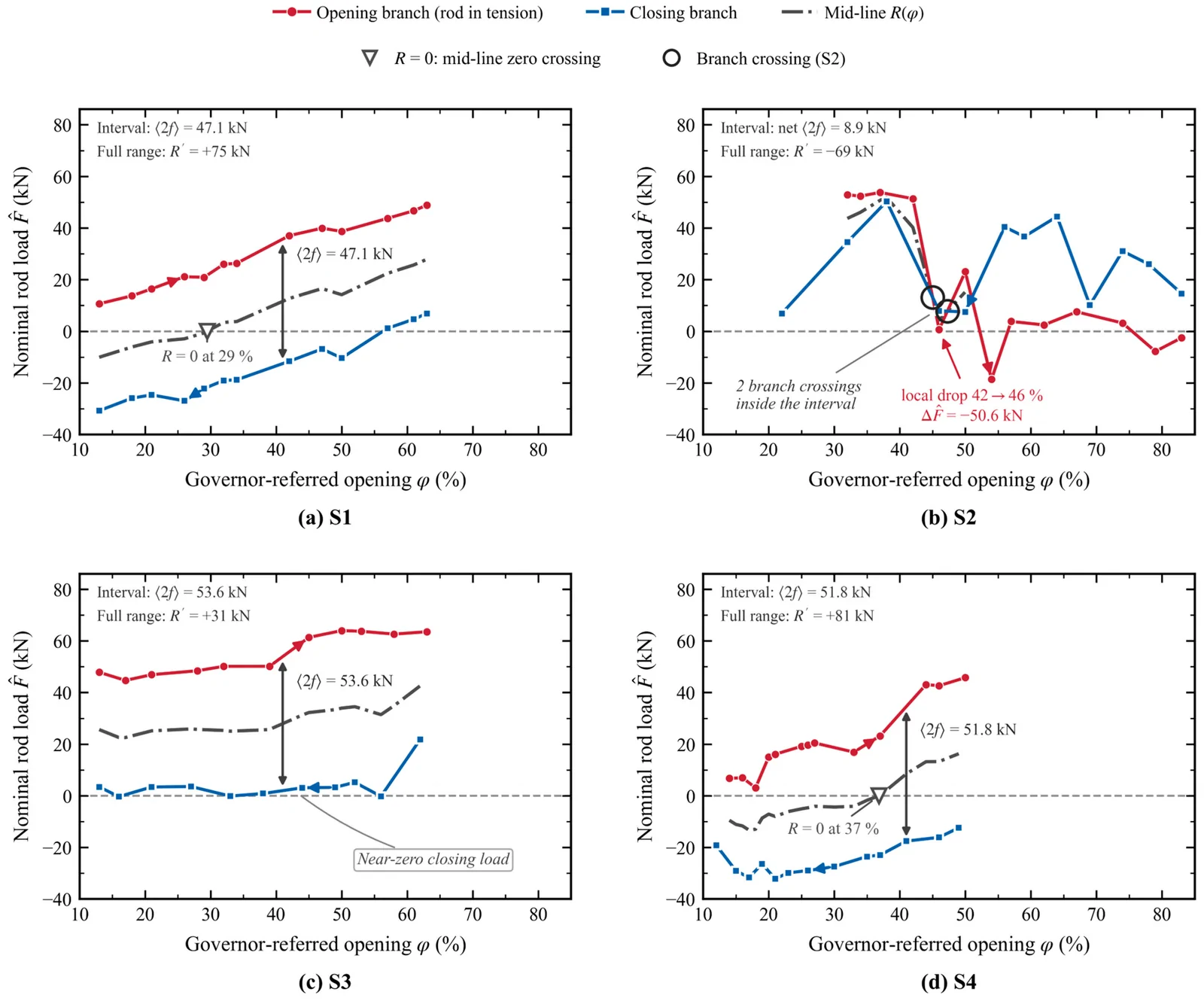
Nominal rod load against governor-referred opening for the as-acquired channel configurations (three-channel mean at S1, S3 and S4; pair {1, 2} at S2). Red circles: opening branch; blue squares: closing branch; dash-dotted line: mid-line *R*(*φ*); open triangle: zero crossing of *R*; open circles in (**b**): branch crossings; arrowheads on the branches indicate the direction of traverse. The double arrow marks the interval mean separation on 32–50%. Corner annotations give the interval mean separation ⟨2*f*⟩ and the full-range midline gradient *R*′, in kN with respect to relative opening; the latter is a reference value and does not enter Equation (10). At S2 the annotated ⟨2*f*⟩ is a net signed mean over crossing branches and is not a dissipation measure.

Figure 3 shows the records as acquired, constructed from every channel that survived in service. The screening reported below subsequently localises a discordant channel at S3, so the values tabulated in Section 3.2 and Section 3.4 differ from the annotations of Figure 3 at that actuator; the difference is stated where it arises.

At S1 the opening branch rises from about 11 kN at 13% to about 49 kN at 63%, while the closing branch runs from about −31 kN to about 7 kN; the branches are nearly parallel and the interval mean separation is 47.1 kN. At S4 the arrangement is similar: the opening branch runs from about 7 kN to 46 kN and the closing branch lies between about −32 and −12 kN, with an interval mean separation of 51.8 kN. At S3 the closing branch remains within a few kilonewtons of zero up to 56% opening, while the opening branch is the highest of the four at 45–64 kN, so the whole loop lies above zero; the interval mean separation is 53.6 kN for the as-acquired three-channel mean. At all three actuators *N*_x_ = 0, so the loops are traversed in the conventional sense throughout the interval: the opening branch lies above the closing branch, and the loop is traversed clockwise in the load–opening plane, as shown by the arrowheads in Figure 3a,c,d and illustrated schematically in Figure 2a.

The mid-line crosses zero at 29% opening at S1 and at 37% at S4 and does not cross at S3. If the bridges were balanced with the rod unloaded, these crossings locate the opening at which the net load transmitted to the regulating ring reverses sense, and S3 transmits a net pulling load throughout its traverse. Because this reading depends on the absolute zero of the load, it is reported descriptively and is not used in any subsequent inference.

S2 differs qualitatively. Its opening branch begins near 53 kN at 32%, holds that level to about 42%, and falls to about 1 kN by 46%, a change of −50.6 kN across a single step. The closing branch rises to about 50 kN near 38% and falls steeply thereafter, so the branches intersect twice within the interval, at approximately 45% and 47%, giving *N*_x_ = 2. Above 50%, the branches alternate with no consistent ordering. Because *N*_x_ ≠ 0, the dissipation indices are undefined for S2, whose loop area would be a net signed quantity.

### 3.2. Descriptors on the Comparison Interval and Window Dependence

The three reference actuators return *H*_D_ between +0.72 and +0.84 and S2 returns +0.17 (Table 3). Taken at face value, the signed separation describes S2 as a loop of conventional sign and about one quarter of the normalised width of the others. The interval loading gradient describes a different situation. The reference actuators return *K*_D_ = +91, +112 and +191 kN, so the rod load rises with opening at each, whereas S2 returns −310 kN, reversed in sign and larger in magnitude than any reference value, with a Type A standard uncertainty of ±101 kN reflecting the scatter of that record. Even allowing for this uncertainty, S2 lies 284 kN, about 2.8 of its own standard uncertainties, below the lower limit of the Screen B tolerance band.

The two descriptors also differ in their dependence on the evaluation window. At S2, *H*_D_ = +0.17 on the comparison interval and −0.20 on the full traversed range; since the full range includes the region later refused by Screen A, this sign change illustrates the fragility of *H*_D_ at a record containing crossings rather than establishing a property of the machine. *H*_D_ is a ratio of a mean separation to a pooled load range, and at a record with crossings the two change with the window in ways that need not compensate. Recomputed on the full traversed range and on windows shifted and widened about Equation (11), *K*_D_ remains negative at S2 and positive at the three reference actuators, although its magnitude varies. Fitting the branches to the recorded points instead of the uniform grid shifts *K*_D_ at S2 from −310 to −241 kN and leaves the reference actuators between +83 and +187 kN. The sign pattern and the separation of S2 from the reference values therefore survive both the choice of window and the choice of weighting, and the screening quantity adopted in Screen B is *K*_D_ rather than *H*_D_. The leave-one-point-out recomputation gives the same picture: removing any single recorded point moves *K*_D_ within 81–97 kN at S1, 92–120 kN at S3 and 169–196 kN at S4, and within −411 to −160 kN at S2, which therefore remains below the lower limit of the Screen B tolerance band whichever point is omitted. *N*_x_ remains zero at the three reference actuators, whereas at S2 it varies between 0 and 2, as expected of a record dominated by a single excursion.

### 3.3. Validity Screening of All Records

The screens are applied to every record, not only to the record that appears anomalous: a screen that has not been exercised on data believed to be sound has no measured specificity, and a screen applied only where a fault is suspected can only confirm the suspicion. All 21 combinations of branch segment and channel pair available in the campaign are evaluated: eighteen at the three-channel actuators (three pairs on each of two branches) and three at S2, where the opening branch of the single surviving pair is divided at 50% into the segment inside the comparison interval and the segment above it, in addition to the closing branch.

#### 3.3.1. Screen A at the Three-Channel Actuators

**Figure 4:**
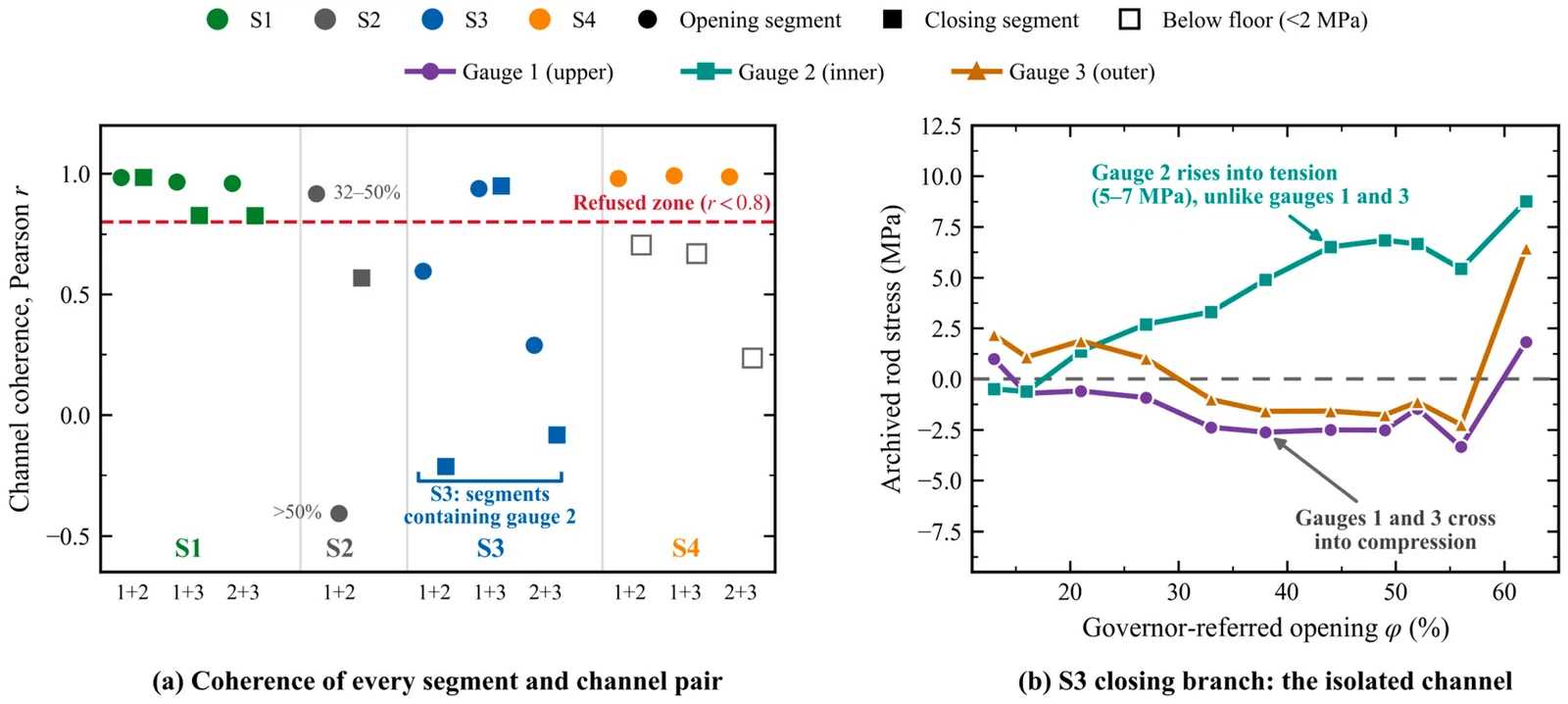
Channel coherence. (**a**) Pearson correlation of the archived stresses for every channel pair and branch segment, grouped by actuator. Circles: opening segments; squares: closing segments; hollow markers: segments below the 2 MPa applicability floor, which are not judged; dashed red line: threshold *r* = 0.8. At S2, the single pair {1, 2} contributes two opening segments, inside the comparison interval (32–50%, *r* = 0.92) and above it (>50%, *r* = −0.41), as well as one closing segment (*r* = +0.57); see also Figure 5a. (**b**) Archived stresses of the three channels on the closing branch at S3: gauge 2 rises into tension (5–7 MPa), whereas gauges 1 and 3 enter compression.

Of the eighteen combinations at the three-channel actuators, three fall below the applicability floor, namely the three closing-branch pairs at S4, in which at least one channel has an archived stress range of only 1.19–1.95 MPa (Figure 4a). Of the fifteen judged, eleven pass and four fail. At S1, every judged combination returns *r* ≥ 0.83 and at S4 every judged combination returns *r* ≥ 0.98. All four failures are at S3 and all contain gauge 2: on the opening branch the pairs {1, 2} and {2, 3} return *r* = +0.60 and +0.29, and on the closing branch −0.21 and −0.08, whereas the pair {1, 3} returns +0.94 and +0.95. Sweeping the correlation threshold from 0.6 to 0.8 and the applicability floor from 2 to 4 MPa leaves the same four failures and no others; indeed, any threshold between the largest failing value (+0.60) and the smallest passing value (+0.83) returns the same verdicts. Omitting the floor adds the three S4 closing-branch combinations, which are the only outcomes that change under any variation in the criteria, as expected of segments that carry little signal rather than adverse signal.

Because *r* rests on eleven to thirteen points at the three-channel actuators, the verdicts were also recomputed with each point removed in turn. The four S3 failures remain failures (largest leave-one-out value +0.71), and every judged combination at S4 and on the opening branch at S1 remains at or above +0.95. The only marginal case is the closing branch at S1, where the two pairs containing gauge 3 return *r* = +0.83 and fall to +0.79 and +0.78 when the most influential point is removed; gauge 3 at S1 therefore lies at the margin of Screen A on that branch. Carrying S1 forward on the pair {1, 2} instead of its three-channel mean would change *w*_L_ from 8.48 to 9.16 kJ/m and *G* from 17 to 16 kN, which would set the cross-actuator coefficient of variation in *w*_L_ at 18.0% and leave the grouping by *G* unchanged. The three-channel mean is retained because both pairs pass at the stated threshold, and the outcome of the alternative is stated so that the reader can weigh it.

The disagreement at S3 is therefore localised to gauge 2. Figure 4b shows its form on the closing branch. Gauges 1 and 3 are in compression above about 16% and 30% opening, respectively, and then run roughly parallel, whereas gauge 2 rises to 5–7 MPa of tension over the same range. Two readings are consistent with this pattern. Under the first, gauge 2 has drifted or partially lost its bond. Under the second, gauge 2 is reporting a genuine bending field: gauges 2 and 3 are diametrically opposed, and on the closing branch they move in opposite senses while their mean, which by Equation (3) is the axial stress, remains within about 3 MPa of zero up to 56% opening. This is the signature of a bending field that grows with opening under a small and nearly constant axial load, which a change in linkage geometry with ring rotation could produce, and which violates the load-proportional bending assumption adopted for the screen without any gauge being faulty. The opening branch weighs somewhat towards the first reading, since the rod load along that branch changes by about 19 kN and the pairs containing gauge 2 still return low coherence, but the records cannot decide between the two.

The screen’s rule is to carry forward the coherent pair, and S3 is therefore reported on {1, 3}. Because {1, 3} is an orthogonal pair that retains bending at half weight, whereas under the bending reading the appropriate axial estimator would be {2, 3}, the effect of this choice on the dissipation indices is quantified explicitly with those indices below. S1 and S4 are carried forward on their three-channel means.

#### 3.3.2. Screen A at the Two-Channel Actuator

**Figure 5:**
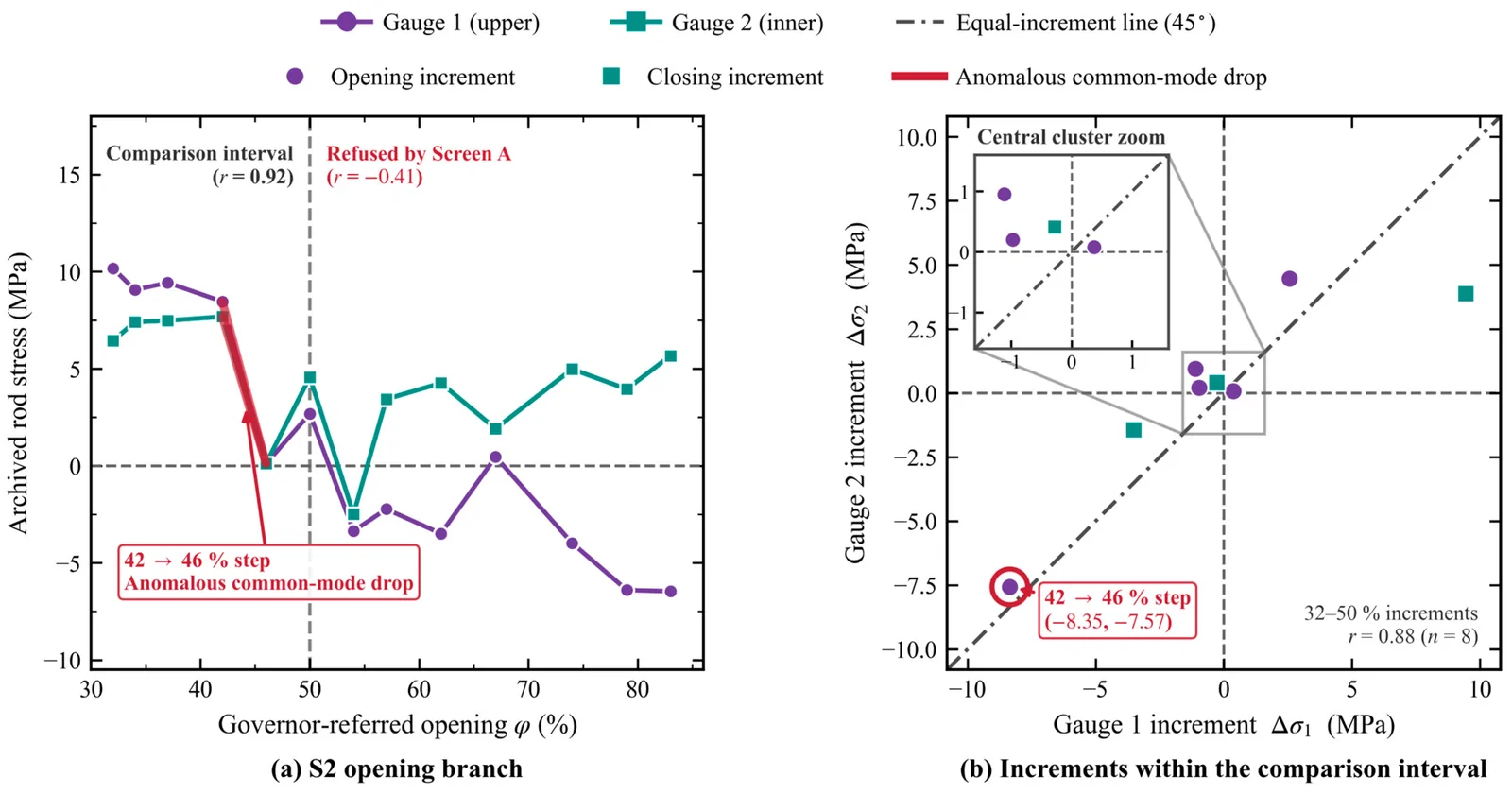
Channel agreement at S2. (**a**) Archived rod stress from the two surviving gauges along the opening branch; the vertical dashed line at 50% separates the comparison interval (*r* = 0.92) from the segment refused by Screen A (*r* = −0.41). (**b**) Increments of the two channels between adjacent recorded openings within 32–50%, five on the opening branch (circles) and three on the closing branch (squares), with the equal-increment line; the circled point is the 42–46% step and the inset enlarges the four increments nearest the origin.

Within the comparison interval, the two surviving channels at S2 move together on the opening branch, beginning at 10.2 and 6.4 MPa at 32%, running approximately in parallel to 42% and falling together to near zero by 46%, with *r* = 0.92 on six points (Figure 5a). Above 50%, they diverge: gauge 1 descends to about −6.5 MPa at 83% while gauge 2 rises to about 5.7 MPa, giving *r* = −0.41 on seven points, and this segment fails Screen A. The closing branch also fails, with *r* = +0.57 over a stress range of 5.1 MPa, well above the floor. Only the opening branch below 50% passes, and because the closing branch enters 2*f*, *H*_D_ and *N*_x_, none of the loop descriptors at S2 rests on a fully coherent record.

Figure 5b examines the manner in which the interval segment passes. The correlation of the eight increments is *r* = 0.88, but it is dominated by the 42–46% step, at Δ*σ*_1_ = −8.35 MPa and Δ*σ*_2_ = −7.57 MPa, which lies close to the equal-increment line; the remaining increments are small and scattered, and one closing increment lies well away from the line. The coherence of small increments at S2 is therefore not established, but the large event is common to both channels. A debonding or bridge fault on one gauge would have placed that point far from the diagonal, so single-gauge failure is excluded as its explanation. The remaining candidates are common-mode: a disturbance in the shared part of the measurement chain; a slip or clearance take-up in the load path, including a partial release of a friction-type or shear-link overload protection in the guide-vane linkage; a fault in the abscissa (Screen D); or a transient jam of the digital valve spool, which would advance the actuator in a discrete lurch and unload the rod at both channels together. The last candidate has precedent in the fault history of this station, which records repeated spool jamming caused by debris from a fatigued filter screen at the valve pressure port [6], but those events are not dated to the present test.

#### 3.3.3. Screens B and C

**Figure 6:**
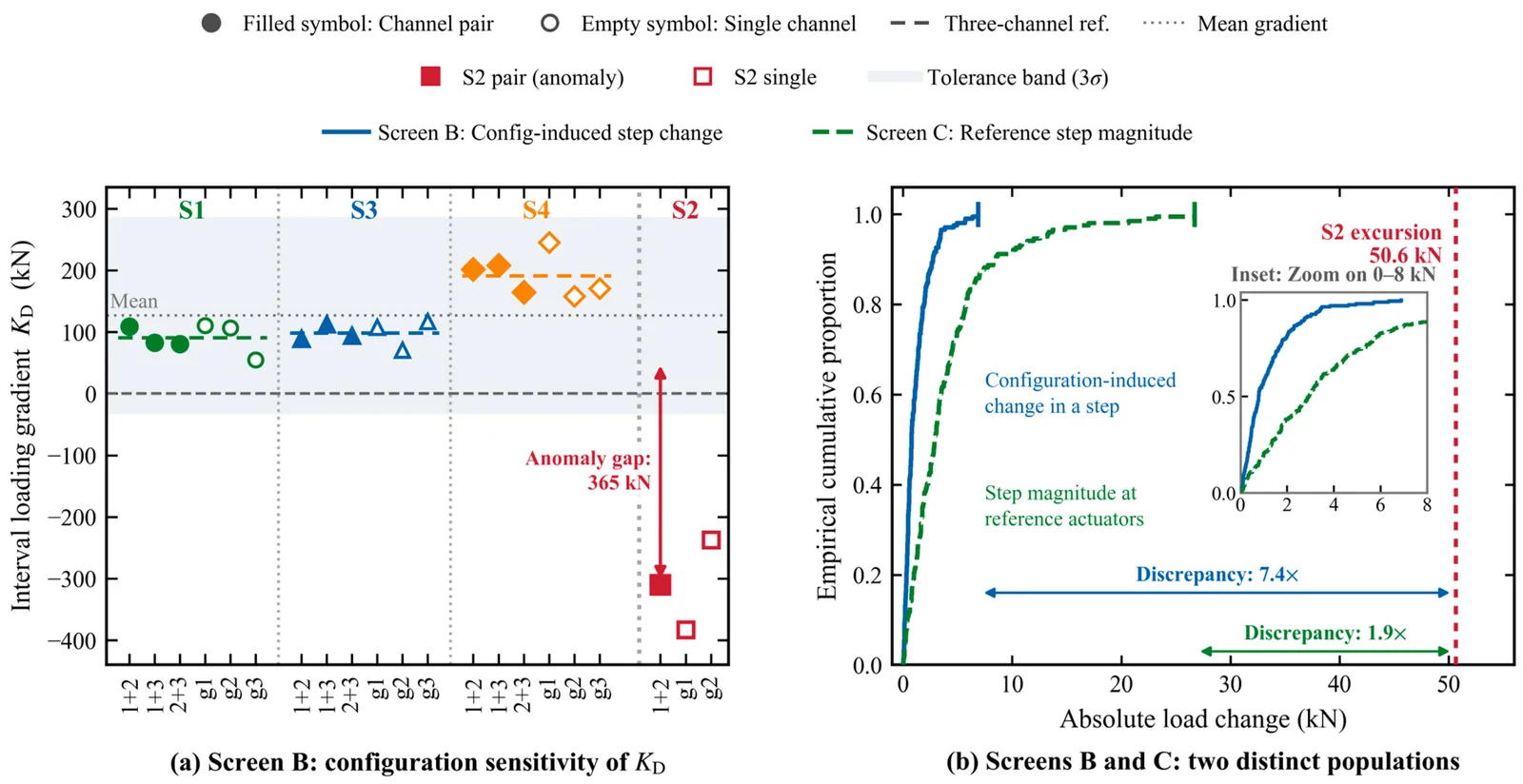
Screens B and C. (**a**) *K*_D_ on 32–50% for every channel configuration at the three reference actuators (filled: pair; open: single channel; dashed line: three-channel value of that actuator) and for the surviving pair and single channels at S2. The shaded band is mean ± 3*s* of the eighteen plotted reference configurations, [−31, 285] kN; the band used for the verdict, which excludes the three S3 configurations containing gauge 2, is [−26, 296] kN and is indistinguishable at this scale. The dotted horizontal line is the mean of the eighteen plotted configurations. (**b**) Empirical cumulative distributions of the change that a channel configuration induces in a recorded step (Screen B) and of the magnitude of the steps themselves at the reference actuators (Screen C), with the S2 excursion marked; the inset enlarges 0–8 kN. In (**a**), dotted vertical lines separate the actuators.

The fifteen reference configurations of Screen B return *K*_D_ between 55 and 245 kN, all positive, with mean 135 kN and standard deviation 54 kN, giving the tolerance band [−26, 296] kN (Figure 6a). All fifteen lie inside the band, and a leave-one-out evaluation, in which each configuration is tested against a band rebuilt from the other fourteen, refuses none. Retaining the three S3 configurations that contain gauge 2 changes the band to [−31, 285] kN and leaves this outcome unchanged. The in-sample specificity is therefore 15/15. This figure should not be over-read: even for fifteen independent trials the one-sided 95% upper bound on the false-positive rate would be about 18%, and the configurations are not independent, so the bound implied by three reference actuators is considerably weaker.

The configuration {1, 2}, which is the one that survived at S2, returns 109 and 202 kN at S1 and S4, close to their three-channel values, so reducing a reference record to this pair displaces *K*_D_ by no more than 18 kN and does not approach zero. S2, on the same pair, returns −310 kN, 365 kN below the lowest reference value (standardised distance 8.3, descriptive only). Its single channels return −383 and −237 kN, both negative and both outside the band, so the anomaly is not carried by one channel that might be discarded in favour of the other. S2 was also traversed at an estimated rate roughly twice that of S1 and S3. A rate difference of this size could alter the width of the loop, but it is not expected to reverse the sign of the loading gradient; it is recorded as a second respect in which S2 is not directly comparable with the reference actuators.

Figure 6b separates the two step populations. The change that a pair configuration induces in a recorded step has a maximum of 6.9 kN over the 204 combinations of three channel pairs and 68 recorded steps at the reference actuators. The S2 excursion of 50.6 kN is 7.4 times that maximum, so the loss of a channel cannot produce it. This population includes the two S3 pairs containing gauge 2; excluding them leaves the maximum unchanged. The magnitude of the steps themselves, over the same 204 pair–step combinations, reaches 26.7 kN, and the S2 excursion is 1.9 times this maximum. It therefore does not reach the three-times criterion of Screen C, which corresponds to a detection limit of 80 kN on this population, and Screen C does not refuse S2. This outcome is reported rather than repaired, since lowering the factor until the record is refused would turn the criterion into a description of the record instead of a test of it. Screen C thus contributes only the observation that the excursion is unusually large, and its sensitivity is correspondingly modest.

#### 3.3.4. Screen D and Verdicts

The archived opening sequence at S2 is strictly monotonic within each branch, with steps of 2–10% and no isolated outlier at the openings where the ordinate anomalies occur, so the abscissa passes the checks that the archive permits. The stronger cross-checks against commanded opening and unit power could not be performed. The check is nonetheless necessary: the fleet fault history records a loosened guide-vane transducer connector at S1 and repeated faults of the guide-vane opening sensor at S3 [6], neither contemporaneous with the tests.

S1 and S4 pass on their three-channel means, and S3 passes with gauge 2 localised and set aside. S2 is refused for dissipation reporting on the basis of Screens A and B, supported by the branch crossings, which are a direct consequence of the 42–46% excursion rather than independent evidence. The verdict concerns the record, not the machine: if the excursion is a hydraulic or mechanical event rather than a measurement event, the record is unfit because it is not quasi-static over the interval, not because a channel misreported. Had the screen not been applied, S2 would have returned a loop area of 1.6 kJ/m computed from a separation that changes sign twice, about one sixth of the reference values, and would have been read as an actuator of unusually low dissipation.

### 3.4. Dissipation Indices of the Records That Pass

**Figure 7:**
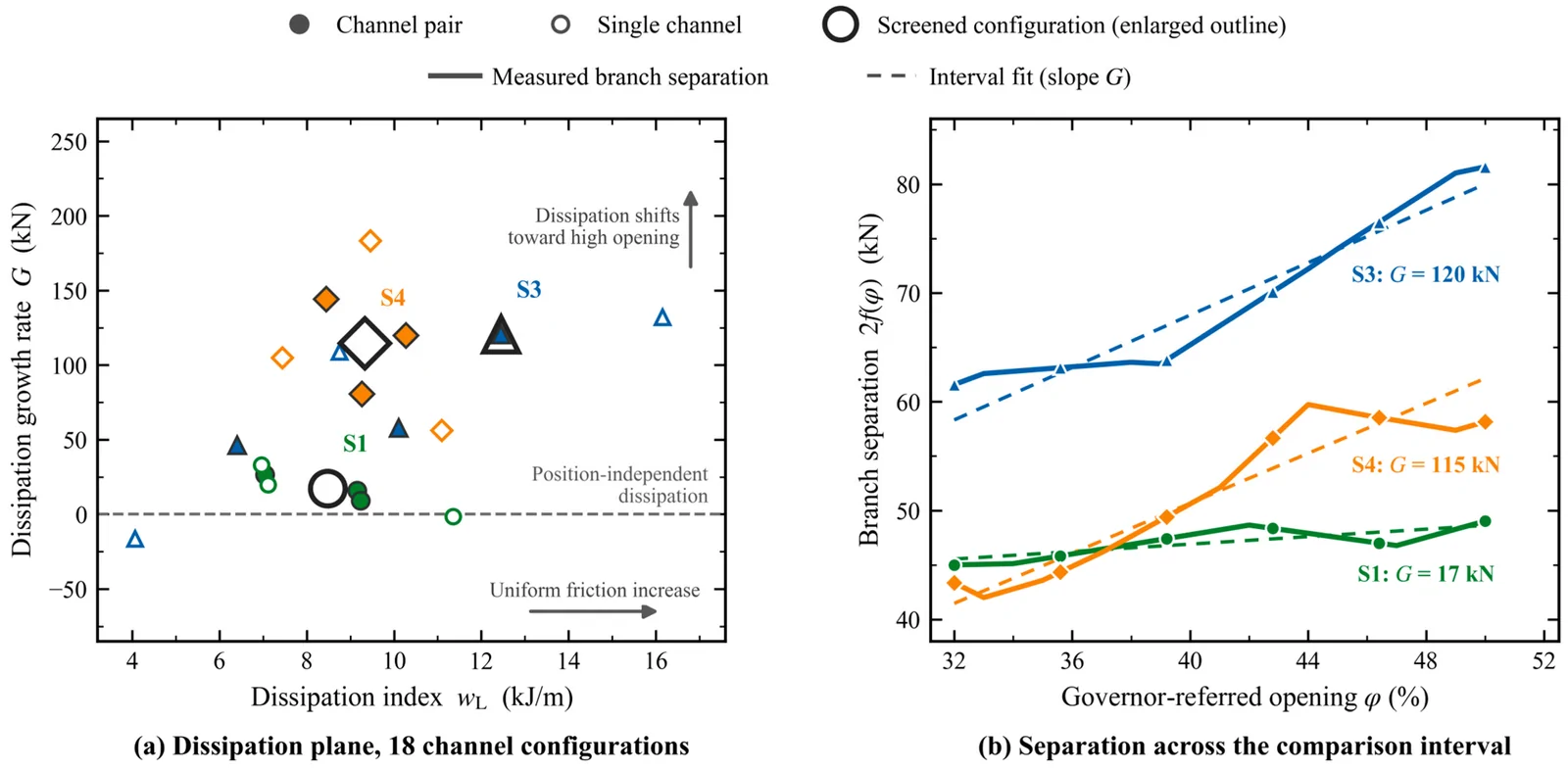
(**a**) Dissipation plane: every channel configuration at the three reference actuators placed at its (*w*_L_, *G*), including the three S3 configurations containing gauge 2 (filled: pair; open: single channel). The enlarged outlined markers are the configurations carried forward: the three-channel mean at S1 and S4 and the pair {1, 3} at S3. (**b**) Interval branch separation 2*f*(*φ*) for the carried-forward configurations, with the least-squares fit whose slope is *G*. *G* is expressed in kN and *w*_L_ in kJ/m.

The dissipation indices of the three records that pass are *w*_L_ = 8.48 ± 0.37, 12.45 ± 0.50 and 9.33 ± 0.50 kJ/m at S1, S3 and S4 (Table 4; combined standard uncertainties, *k* = 1), a coefficient of variation of 20.8% about a mean of 10.1 kJ/m. The difference of 3.97 kJ/m between S3 and S1 is about six combined standard uncertainties. Removing any single recorded point changes these values by at most 0.4 kJ/m (ranges 8.32–8.63, 12.31–12.85 and 9.11–9.50 kJ/m). Evaluated instead on the three-channel mean at every actuator, as would be natural without screening, they are 8.48, 9.65 and 9.33 kJ/m, a coefficient of variation of 6.6%. The whole difference arises at S3, from the channel configuration carried forward. The two pair configurations at S3 that contain gauge 2 return *w*_L_ = 6.4 kJ/m for {1, 2} and 10.1 kJ/m for the diametrically opposed pair {2, 3}, which is the appropriate axial estimator under the bending reading of gauge 2 discussed above (Figure 7a); substituting them for S3 gives coefficients of variation of 18.6% and 8.8%, respectively. The cross-actuator dispersion of *w*_L_ therefore lies anywhere between 6.6% and 20.8% according to how the three channels of a single actuator are combined. A regularity that appears or disappears according to which channels of one actuator are averaged cannot be reported as a cross-station property.

Figure 7a confirms that *w*_L_ is not robust to channel configuration. Among pair configurations, it spans 7.0–9.2 kJ/m at S1, 6.4–12.4 kJ/m at S3 and 8.4–10.3 kJ/m at S4, and admitting single channels widens the range to 4.1–16.1 kJ/m. The configuration effect at one actuator is thus comparable with, or larger than, the differences between actuators, and *w*_L_ can support tracking of one actuator through an unchanged chain and configuration but not absolute comparison between machines.

The growth rate behaves differently. The carried-forward values are *G* = 17 ± 21, 120 ± 34 and 115 ± 29 kN, all positive, so at each actuator the loop widens as the vanes open (Figure 7b). At S1, the separation grows by 3.1 kN across the interval, 7% of its mean, which is not distinguishable from zero within its standard uncertainty, so the direction-dependent resistance is nearly position-independent. At S3 and S4, it grows by 21.7 and 20.7 kN, 31% and 40% of the respective means, indicating a substantial position-dependent component. Because the measured load excludes the servomotor seals, candidate mechanisms lie in the driven mechanism: an opening-dependent reaction at the regulating-ring guides or vane-stem bushings associated with the changing linkage geometry, and an opening-dependent contribution of the vane hydraulic torque. The present measurements do not discriminate between them.

The distinction between S1 and the other two actuators in *G* is robust to channel configuration within stated limits. Every configuration at S1 returns *G* below 35 kN, and every configuration at S3 and S4 that excludes gauge 2 at S3 returns *G* above 50 kN (Figure 7a). The three S3 configurations containing gauge 2 return *G* = 46 kN for {1, 2}, 58 kN for {2, 3} and −16 kN for gauge 2 alone, so the grouping survives the loss of any channel that passes Screen A, and it also survives the bending reading of gauge 2, since both pairs containing gauge 2 return *G* above the S1 range; it fails only if the localised channel is used by itself. S3 and S4 are not separable from each other in *G*. In terms of the combined standard uncertainties, the carried-forward value at S1 differs from those at S3 and S4 by 103 ± 40 kN and 98 ± 36 kN, that is, by about 2.6 and 2.7 standard uncertainties, and removing any single recorded point leaves *G* between 3 and 35 kN at S1, 100 and 131 kN at S3 and 92 and 129 kN at S4. The grouping is therefore supported by the single traverse, although not at a level that would exclude a substantial run-to-run variation.

## 4. Discussion

### 4.1. What the Screen Can and Cannot Decide

The campaign contains both cases that a redundant strain section can present. At S3, three channels allowed a disagreement to be localised to one gauge, and the other two actuators with three channels passed; at S2, two channels allowed a disagreement to be detected but not attributed. This asymmetry is the practical argument for fitting three gauges rather than two. The S3 case also shows the limit of a 0°/90°/180° layout. Because such a layout is exactly determined under general bending, a localised disagreement is a violation of the load-proportional bending assumption, which a faulty gauge and a geometry-dependent bending field produce equally well. A fourth gauge at 270° removes this ambiguity at negligible cost: the two diametrically opposed pairs then provide two independent estimates of the axial strain, (*ε*_1_ + *ε*_4_)/2 and (*ε*_2_ + *ε*_3_)/2, whose agreement is a genuine redundancy check under an arbitrary linear bending field, and the bending components are recovered from the pair differences.

The specificity of the screen was measured on reference data rather than assumed, which is the main methodological point of applying it symmetrically. Its sensitivity, however, has been characterised only for Screen C, through a detection limit of 80 kN. The detection power of Screens A and B against drift, gain loss or partial debonding of a single channel has not been quantified. This could be done without new field data by injecting synthetic faults of controlled magnitude into the reference records and recording the detection rate of each screen, and it is the most direct extension of the present analysis. Where continuous time series are archived, the elementary screens should also be replaced or supplemented by established residual-based tests [20,21,22].

The screen also changed a headline result. Without it, the three dissipation indices would have been reported as agreeing within a coefficient of variation of 6.6%, and S2 would have been reported as an actuator of unusually low dissipation. Neither conclusion survives screening. Depending on the unresolved reading of gauge 2, the screened dispersion is represented by the {1, 3} configuration at S3, giving a coefficient of variation of 20.8%, or by the {2, 3} configuration, giving 8.8%; in every case it depends on a configuration choice at a single actuator, which is itself the finding.

### 4.2. Physical Reading of the Indices and Condition Tracking

Because the gauged section lies outside the cylinder, *w*_L_ and *G* describe the resistance of the driven mechanism and the direction dependence of the vane loading rather than seal friction in the servomotor. This bears on how the indices should be used. Seal wear and internal leakage in hydraulic cylinders are diagnosed from pressure and flow signatures [35,36], and would not be visible in the rod-load loop. Changes in the regulating-ring guides, the vane-stem bushings and the linkage, on the other hand, would be. For friction compensation in the governor, which requires the total resistance seen by the servo loop [13,37], the rod-load loop is a lower bound, and chamber pressures are needed to complete it.

Two further restrictions follow from the definition of the indices. The indices describe a traverse of the full comparison interval, whereas regulating corrections are small and trace minor loops inside it, so *w*_L_ does not give the energy dissipated per regulating reversal; minor-loop characterisation would be required for that purpose. And since *w*_L_ is moved by channel configuration more than it differs between actuators, the practical use of the indices is to track one actuator against itself. For one actuator instrumented through one chain with a frozen channel configuration and re-tested at successive outages, the scale error *c* is common to all tests, so displacements in the (*w*_L_, *G*) plane are known more accurately than positions in it. A tentative reading of such displacements is that movement in *w*_L_ at approximately constant *G* indicates a uniform increase in resistance, for example through lubricant degradation in the ring guides or bushings, whereas movement in *G* at approximately constant *w*_L_ indicates a redistribution of resistance towards high opening, pointing to a position-dependent mechanism in the linkage. This interpretation is offered as a framework to be tested, not as a validated diagnostic chart. The branch separation is also, within the loading trend, the rod load range of a full reversal, so a growing separation implies a larger cyclic load range on the rod and its connections [10,38].

### 4.3. Recommendations for Instrumentation and Reporting

Four recommendations follow for field campaigns of this kind. First, a strain section on a push–pull rod should carry four gauges at 90° intervals, or, where only three can be fitted, the diametrically opposed pair should be the most robustly protected, since it alone cancels a bending component exactly; at S2 the surviving pair was an orthogonal one. Second, the bridge balancing state should be recorded, preferably with the rod unloaded, so that absolute load levels and midline crossings become interpretable; chamber pressures should be recorded where the total actuator friction is of interest. Third, continuous time series should be archived alongside reduced points, and characterisation manoeuvres should be repeated and performed at two or more traverse rates, which would provide a repeatability estimate and allow rate-independent and rate-dependent contributions to be separated [33]. Fourth, field hysteresis and dissipation figures for hydraulic actuators should be published together with an explicit validity screen covering both axes of the loop and applied to every record, or otherwise labelled as unscreened.

### 4.4. Limitations

The results are bounded by the following limitations. The stroke was not calibrated, so *w*_L_ is an energy per unit stroke. Each actuator was characterised in one opening and one closing manoeuvre at a single traverse rate, so no repeatability estimate exists (its potential effect is bounded in Section 2.9) and rate-dependent contributions cannot be separated, and the traverse rates are indirect estimates, with none available for S4. The descriptors are defined on 32–50% of travel with four to six points per branch, and the gradients rest on small fits. The reference population comprises three actuators whose channel configurations are not independent, so the specificity of Screen B is only weakly bounded, and the sensitivity of Screens A and B has not been characterised. The scale error *c* was estimated from nominal specification limits rather than from calibration records, and absolute load levels depend on an undocumented zero state. Finally, the screen establishes that the S2 record cannot support a dissipation figure without identifying the cause of the 42–46% event, and it localises a disagreement at S3 without deciding between a gauge fault and a geometry-dependent bending field.

## 5. Conclusions

A redundancy-based validity screen for reduced strain-channel records from in-service hydraulic servomotors was developed and applied to every record from four guide-vane servomotors under complementary regulation duty before any load characteristic was reported. The main conclusions are as follows.

(1) A validity screen must be exercised on records believed to be sound. Applied symmetrically, the configuration screen refused none of fifteen reference channel configurations, in sample or under leave-one-out, and the coherence screen returned the same four failures under every threshold and floor examined once an applicability floor of 2 MPa was imposed; under leave-one-point-out, only the two S1 closing-branch pairs containing gauge 3 fell marginally below the threshold. Because the reference configurations are not independent, this specificity is only weakly bounded, and detection sensitivity remains to be characterised.

(2) Three channels at one section permit a disagreement to be localised; two permit it only to be detected. Both cases occurred. At a 0°/90°/180° layout, a localised disagreement cannot be allocated between a gauge fault and a bending field that varies independently of the axial load; a fourth gauge forming a second diametrically opposed pair removes this ambiguity.

(3) A record whose interval loading gradient was −310 kN per unit relative opening, 365 kN below the lowest reference value, and whose channels failed the coherence screen outside the interval and on the closing branch, was refused for dissipation reporting. The associated 50.6 kN excursion was common to both channels and at least 7.4 times the largest change that a channel configuration induced in a step, so single-gauge failure and channel loss are excluded as explanations, although the cause remains unidentified.

(4) Loop energy can be reported without a calibrated stroke through a stroke-free dissipation index in energy per unit stroke. The index was, however, moved by channel configuration at one actuator more than it differed between actuators, and its cross-actuator coefficient of variation ranged from 6.6% to 20.8% according to the configuration carried forward at a single actuator; it supports tracking of one actuator through a frozen configuration, not absolute comparison between machines.

(5) The dissipation growth rate is more robust. Its point estimates differed by a factor of about seven between actuators, a difference of about 2.6–2.7 combined standard uncertainties, and the separation of the actuator with near-position-independent resistance from the other two was preserved for every configuration that excluded the localised gauge. Because the gauged section lies outside the cylinder, both indices describe the driven mechanism and the vane loading rather than servomotor seal friction.

## Figures and Tables

**Table 1:** The four instrumented guide-vane servomotors. The opening-branch range is the span actually traversed; the intersection of these ranges defines the comparison interval, 32–50%, used throughout. The last column gives the number of recorded points inside that interval on each branch.

Actuator	Rated Head (m)	Rated Capacity (MW)	Rod Diameter d (mm)	Strain Channels at Test	Opening Branch Traversed (%)	Points on 32–50% (Open/Close)
S1	130	18.6	80	3 (1, 2, 3)	13–63	5/5
S2	118	15.5	90	2 (1, 2; gauge 3 lost)	32–83	6/4
S3	132	20.6	70	3 (1, 2, 3)	13–63	4/4
S4	91	12.0	102	3 (1, 2, 3)	14–50	5/5

**Table 2:** The four validity screens applied to every record. A record is carried forward for dissipation reporting only if it passes all four screens and satisfies *N*_x_ = 0, i.e., its branches do not cross.

Screen	Quantity Examined	Criterion	Verdict
A	Pearson correlation *r* between the stresses of each channel pair on each branch segment	*r* ≥ 0.8; judged only where the stress range of both channels exceeds 2 MPa	Three channels: discordant channel localised and set aside. Two channels: record refused
B	Interval loading gradient *K*_D_ recomputed for every pair and single-channel configuration	*K*_D_ within mean ± 3*s* of the 15 reference configurations	Record refused if outside the band
C	Largest load increment between adjacent recorded openings	Increment ≤3× the largest step at the reference actuators	Record refused on the interval containing the excursion
D	Recorded opening *φ* within each branch	Monotonic sequence with no discontinuity at openings where load anomalies occur	Record refused if violated

**Table 3:** Hysteresis descriptors on the comparison interval 32–50%. S3 is evaluated on the pair {1, 3}, gauge 2 having been localised by Screen A; S1 and S4 are evaluated on their three-channel means and S2 on its surviving pair. The last column is the Type A standard error of *K*_D_ from the residuals of the least-squares fit to the recorded points on the interval, a lower bound on the combined uncertainty; adding the Type B scale component in quadrature leaves it unchanged at the precision shown, and neither includes repeatability.

Actuator	Channels Used	*H*_D_ (–)	*K*_D_ (kN)	Type A s.e. of *K*_D_ (kN)	*N*_x_ (–)	⟨2*f*⟩ (kN)
S1	{1, 2, 3}	+0.80	+91	±15	0	47.1
S2	{1, 2}	+0.17	−310	±101	2	8.9 ^a^
S3	{1, 3}	+0.84	+112	±34	0	69.2
S4	{1, 2, 3}	+0.72	+191	±27	0	51.8

^a^ Net signed mean over two crossing branches; not a dissipation measure.

**Table 4:** Dissipation indices on the comparison interval from Equations (9) and (10), with both branch gradients restricted to the interval. S3 is evaluated on the pair {1, 3}. Values are given with approximate combined standard uncertainties (*k* = 1), which do not include repeatability (Section 2.9). The last two columns give the growth of the separation across the interval in absolute terms and relative to the interval mean separation; the latter is independent of the scale error *c*. n.d.: not defined, since *N*_x_ ≠ 0.

Actuator	*w*_L_ (kJ/m)	*K*_c_ (kN)	*G* (kN)	*G*Δ*y* (kN)	*G*Δ*y*/⟨2*f*⟩ (–)
S1	8.48 ± 0.37	+74 ± 15	17 ± 21	3.1 ± 3.8	0.07 ± 0.08
S2	n.d.	n.d.	n.d.	n.d.	n.d.
S3	12.45 ± 0.50	−8 ± 5	120 ± 34	21.7 ± 6.1	0.31 ± 0.09
S4	9.33 ± 0.50	+76 ± 10	115 ± 29	20.7 ± 5.2	0.40 ± 0.10

## Data Availability

The reduced load–opening records supporting the findings of this study are available from the corresponding author upon reasonable request; station names are withheld for confidentiality.

## Nomenclature

The following symbols are used in this manuscript.

**Symbol**	**Description**	**Unit**
*A*	Nominal cross-sectional area of the rod, *πd*^2^/4	mm^2^
*A*_L_	Interval loop area in the plane of load and relative opening	kN
*b*	Offset of the reported rod load fixed by bridge balancing	kN
*C*	Set of strain channels used	–
*c*	Multiplicative scale error of the reported rod load	–
*d*	Rod diameter	mm
*E*	Elastic modulus of the rod material	GPa
$F,\;\hat{F}$	True and nominal (reported) rod load, positive in tension	kN
${\hat{F}}_{o}$, ${\hat{F}}_{c}$	Nominal rod load on the opening and closing branches	kN
*f*	Half-width of the loop; 2*f* is the branch separation	kN
*G*	Dissipation growth rate, d(2*f*)/d*y* = *K*_o_ − *K*_c_	kN
*H*_D_	Signed normalised separation	–
*K*_D_ ≡ *K*_o_	Interval loading gradient of the opening branch, $d{\hat{F}}_{o}$/d*y*	kN
*K*_c_	Interval gradient of the closing branch	kN
*k*	Coverage factor	–
*L*	Full actuator stroke	m
*N*_x_	Branch crossing count within the comparison interval	–
*n*	Number of recorded points per branch in the interval	–
*R*, *R*′	Mid-line of the loop and its least-squares gradient with respect to *y*	kN
*r*	Pearson correlation coefficient between two channels	–
*s*	Standard deviation of the reference values of *K*_D_	kN
*s*_res_	Residual standard deviation of an interval fit	kN
*u*(·)	Standard uncertainty	–
*W*	Energy dissipated in one traverse of the interval	kJ
*w*_L_	Stroke-free dissipation index, *W*/*L*	kJ/m
*x*	Rod displacement	m
*y*	Relative opening, *φ*/100	–
Δ*φ*, Δ*y*	Width of the comparison interval in opening and relative opening	%, –
*ε*_0_, *ε*_i_	Axial strain; surface strain of channel *i*	µε
*η*	Zero-mean random component of the reported rod load	kN
*θ*	Circumferential position of a gauge	°
*κ*_x_, *κ*_y_	Curvature components of the bending field	1/m
*σ*_i_, $\bar{\sigma }$	Stress of channel *i*; nominal axial stress	MPa
*φ*	Guide-vane opening reported by the governor feedback transducer	%
[*φ*_1_, *φ*_2_]	Comparison interval (32–50%)	%
⟨·⟩	Mean over the comparison interval	–
